# Identification and In Vivo Validation of Unique Anti-Oncogenic Mechanisms Involving Protein Kinase Signaling and Autophagy Mediated by the Investigational Agent PV-10

**DOI:** 10.3390/cancers16081520

**Published:** 2024-04-16

**Authors:** Son Tran, Patrick Sipila, Satbir Thakur, Chunfen Zhang, Aru Narendran

**Affiliations:** Department of Oncology, University of Calgary, Calgary, AB T2N 1N4, Canada

**Keywords:** PV-10, rose bengal sodium, solid tumors, novel therapeutics, intralesional, systemic

## Abstract

**Simple Summary:**

Novel therapeutics are urgently needed for high-risk and refractory solid tumors. Clinical studies have demonstrated the safety and efficacy of PV-10 (10% rose bengal sodium) by intralesional injection in skin cancer. However, this agent has not yet been evaluated for the treatment of various adult solid tumors. The aim of our study was to test PV-10 in breast, colorectal, head and neck, and testicular cancers. Using a combination of in vitro and in vivo experiments, we found that PV-10 exhibits anti-cancer activity against a panel of human cell lines derived from these tumors. Our results support further clinical development of PV-10 for the treatment of solid tumors in adults.

**Abstract:**

PV-10 is a 10% formulation of rose bengal sodium that has potent immunotherapeutic and anti-cancer activity against various tumors, including metastatic melanoma and refractory neuroblastoma. Currently, PV-10 is undergoing clinical testing for refractory metastatic neuroendocrine cancer and melanomas. However, preclinical investigation of PV-10 activity and its mechanisms against phenotypically and molecularly diverse adult solid tumors had not been conducted. In a panel of human cell lines derived from breast, colorectal, head and neck, and testicular cancers, we demonstrated that PV-10 induces cytotoxicity by apoptotic and autophagic pathways involving caspase-mediated PARP cleavage, downregulation of SQSTM1/p62, and upregulation of beclin-1. Treatment with PV-10 also consistently reduced phosphorylation of WNK1, which has been implicated in cancer cell migration and autophagy inhibition. By wound healing assay, PV-10 treatment inhibited the migration of cancer cells. Finally, significant inhibition of tumor growth was also noted in tumor-bearing mice treated with PV-10 by intralesional or systemic administration. In addition to known PV-10-mediated tumor-specific cytotoxic effects, we identified the mechanisms of PV-10 and provide new insights into its effect on autophagy and metastasis. Our data provide essential mechanism-based evidence and biomarkers of activity to formulate clinical studies of PV-10 in the future.

## 1. Introduction

PV-10, a 10% weight/volume (*w*/*v*) formulation of rose bengal sodium (RBS; 4,5,6,7-tetrachloro-2′,4′,5′,7′-tetraiodofluorescein disodium), is a water-soluble, halogenated xanthene dye with photochemical properties that has been studied for uses in ophthalmology, dermatology, and oncology [1]. Developments of various derivatives [2] and drug delivery systems [3] have aimed to improve its physicochemical properties, such as its bioavailability and cell membrane permeability that have limited RBS to intralesional utility [4]. Accordingly, rose bengal lactone (RBL) is a derivative of RBS with reduced photosensitivity and increased lipophilicity [5].

Remarkably, early phase clinical testing has demonstrated efficacy and tolerability by intralesional injection of PV-10 in patients with refractory metastatic melanoma [6,7,8,9,10,11]. In a phase II clinical trial, greater efficacy was observed when patients were treated with PV-10 in combination with radiotherapy, with complete response and overall response rates of 42% and 87%, respectively [8]. Consistent with its known safety profile, clinical studies of PV-10 have reported minimal systemic and severe adverse events. Notably, transient phototoxicity has been reported following intralesional administration of PV-10 in melanoma, possibly due to synergistic effects in combination with another known photosensitizing agent, thiazide diuretics [12]. Thus, photoprotection from sunlight and artificial light may be warranted.

Clinical trials are currently testing intralesional PV-10 as a monotherapy and in combination with immune checkpoint inhibitors for refractory neuroendocrine tumors metastatic to the liver (ClinicalTrials.gov ID: NCT02693067), metastatic cutaneous melanoma (ID: NCT02557321), and primary or metastatic liver cancer (ID: NCT00986661). In addition to skin cancers, intralesional PV-10 has also demonstrated tumor regression in xenograft mouse models with breast [13], colon [14], and pediatric neuroblastoma [15] tumors. Given the established clinical efficacy of PV-10 in melanoma and preclinical data in other cancers via intralesional injection, there is a need to evaluate the therapeutic potential of PV-10 in high-risk and refractory solid tumors via systemic administration.

In the present study, we conducted a preclinical investigation of PV-10 activity against a subset of diverse tumors. First, we confirmed anti-cancer efficacy and induction of apoptosis by PV-10 in vitro against a panel of adult solid tumor cell lines and characterized its effects on protein kinase signaling, autophagy, and cell migration. We then developed a xenograft model to assess the efficacy of PV-10 treatment in vivo and provide the first preclinical data on systemic treatment of solid tumors using PV-10.

## 2. Materials and Methods

### 2.1. Materials

PV-10, a 10% *w*/*v* solution of rose bengal sodium (RBS) in 0.9% saline, and its derivative, RBL, were provided by Provectus Biopharmaceuticals, Inc. (Knoxville, TN, USA) and stored protected from light at room temperature. WNK463 (Selleckchem, Houston, TX, USA) was dissolved in dimethyl sulfoxide (DMSO) and stored at −80 °C. For experiments, the drugs were diluted in Opti-MEM to the indicated concentrations.

### 2.2. Cell Culture

The following cancer cells were used in this study: breast [MCF-7 (age: 69/sex: F), MDA-MB-231 (51/F), and T-47D (54/F)], colorectal HCT-116 (48/M), LoVo (56/M), and T-84 (72/M), head and neck [CAL-27 (56/M), Detroit-562 (unknown/F), FaDu (56/M), and UM-SCC-1 (72/M)], and testicular [NCC-IT (24/M), NTERA-2 (22/M), and TCAM-2 (35/M)] cancer (Table S1). Cell lines were obtained from the American Type Culture Collection (ATCC; Manassas, VA, USA) or generously provided by Dr. Karl Riabowol from the University of Calgary (Calgary, AB, Canada). Cells were cultured in Opti-MEM media containing insulin, other metabolites and trace elements, and supplemented with 10% fetal bovine serum (FBS; Thermo Fisher Scientific, Waltham, MA, USA) and maintained at 37 °C in a 5% CO_2_ humidified incubator.

### 2.3. Cytotoxicity Assays

Cytotoxicity was assessed in a similar range to concentrations previously tested in other cancer cells [14,15,16,17]. Initially, cells were seeded in triplicate in 96-well plates at 5 × 10^3^ cells per well in 100 μL of media. After 24 h, the cells were treated with 3.125–400 µM PV-10/RBL or phosphate-buffered saline (PBS) diluted in 100 µL of Opti-MEM media. After treatment for 96 h, cells were washed twice with PBS and fresh media were added to each well for cell viability assay using the alamarBlue^®^ (alamar blue) reagent (Thermo Fisher Scientific, Waltham, MA, USA) according to the manufacturer’s protocol. Alamar blue reagent was added to 10% volume in each well and incubated at 37 °C for 1–4 h, followed by fluorescence measurements (excitation/emission: 560/590 nm). Half-maximal inhibitory concentrations (IC_50_) were determined by non-linear regression.

### 2.4. Western Blot Analysis

Cells were seeded at 10^6^ cells per well in six-well plates. After 24 h, cells were treated with 50–100 μM PV-10 or PBS for up to 48 h and whole cell lysates were prepared. Protein concentration in lysates was measured using the DC Protein Assay (Bio-Rad, Hercules, CA, USA) according to the manufacturer’s protocol and absorbance measurements at 750 nm. Lysates containing 30 µg of protein in Laemmli sample buffer were resolved by denaturing 7.5–15% polyacrylamide gel electrophoresis (SDS-PAGE) and transferred to nitrocellulose membranes. Membranes were blocked using 5% (*w*/*v*) bovine serum albumin (BSA) in tris-buffered saline with 0.1% (*v*/*v*) Tween-20 (TBS-T) for 1 h. The membranes were then incubated overnight at 4 °C with the following primary antibodies diluted in TBS-T with 5% BSA: anti-poly-(ADP-ribose) polymerase (PARP; 1:1000, 9542S); anti-caspase-3 (1:1000, 9662S); anti-SQSTM1/p62 (1:1000); anti-beclin-1 (1:1000, 3738S); anti-β-catenin (1:1000, 9562S); and anti-β-actin (1:1000, 4967S) (Cell Signaling Technology, Danvers, MA, USA). After incubation with primary antibodies, the membranes were washed three times with TBS-T and incubated with horseradish peroxidase (HRP)-conjugated secondary antibody (1:10,000; 7074S) for 1 h. After washing three times, Western blots were developed in Clarity Western ECL substrate, imaged using the Bio-Rad ChemiDoc™, and analyzed using Image Lab software (Version 6.1, Bio-Rad, Hercules, CA, USA). Protein molecular masses in kilodaltons (kDa) were estimated using the Precision Plus Protein™ Dual Color Standards (Bio-Rad).

### 2.5. Phospho-Kinase Array

The relative phosphorylation level of 37 proteins and total level of 2 other proteins was measured using the Human Phospho-Kinase Array Kit (R&D Systems, Minneapolis, MN, USA) according to the manufacturer’s protocol. Briefly, 10^6^ cells were cultured for 24 h in six-well plates. Cells were treated with 100 μM PV-10 or PBS for 3 h, washed with ice-cold PBS, and cell lysates were collected. The phospho-kinase arrays were incubated with approximately 400 μg of cell lysate overnight at 4 °C. Following washing, the arrays were incubated with HRP-conjugated streptavidin and developed by luminol-based chemiluminescence. Reference spots containing unrelated biotinylated proteins were used to determine equal development.

### 2.6. Wound Healing Assay

Cells were seeded at 10^6^ cells per well in a six-well plate and cultured to full confluency. Two hours prior to scratching, cells were treated with a sublethal dose of 10 μM PV-10 or 1 µM WNK463, compared to the PBS control. Using a P200 pipette tip, cells were scraped in a straight line to form a scratch void of cells. Debris was removed by washing with PBS and fresh Opti-MEM containing 5% FBS was added. Plates were imaged by phase-contrast microscopy using an EVOS FL Auto imaging system (Thermo Fisher Scientific) with 4× magnification at 0 and 24 h following the initial scratch. Wound closure was measured using the “MRI wound healing tool” plugin for ImageJ (Version 2.14, National Institutes of Health, Bethesda, MD, USA), normalized to the initial scratch area at 0 h to generate a percent wound closure.

### 2.7. In Vivo Xenograft Models

All animal procedures were carried out in accordance with the Canadian Council on Animal Care and the National Institutes of Health guidelines on the use of laboratory animals and ethics approval by the University of Calgary Animal Care Committee to study the biology of pediatric and adult tumors and to test novel therapeutic agents in mouse xenograft models. BALB/c and CB17 severe combined immunodeficiency (SCID) female mice (Charles River Laboratories, Wilmington, MA, USA) were housed with a 12:12 h light–dark cycle at 23 °C and 40–60% relative humidity and provided with commercial rodent chow and water ad libitum. To provide insight into systemic tolerability, four BALB/c mice were treated with 0, 50, 100, or 200 mg of RBL per kg of body weight (mg/kg) in 0.1 mL PBS by oral gavage. After two weeks, tissues from the brain, heart, kidney, liver, lung, and spleen were isolated and sectioned for hematoxylin and eosin staining. The slides were independently analyzed by a pathologist at the University of Calgary’s Department of Pathology & Laboratory Medicine that was not associated with the project. For xenograft experiments, 36 SCID mice were randomized into treatment groups. To validate clinically established routes of administration using PV-10, three mice per intralesional treatment group were used to reduce the number of animals. For the oral route of administration using RBL, there were six mice per treatment group. At day 0, six- to eight-week-old mice were subcutaneously injected in the right flank with 5 × 10^5^ FaDu cells or 3 × 10^6^ NTERA-2 cells suspended in 0.1 mL PBS. After tumor cell injection, animals with detectable tumor growth were treated with PBS, PV-10 or RBL. The mice injected with FaDu cells were treated with 0.08 or 0.24 mL of PV-10 per cm^3^ of lesion volume (mL/cm^3^) by intralesional injection. The mice injected with NTERA-2 cells were treated with 110 or 220 mg/kg RBL by oral gavage. Mice in the control group received an equivalent volume of PBS via the respective route of administration. Animals were monitored daily and their tumor areas were measured twice weekly with a vernier caliper until any mouse met the endpoint criteria. The defined experimental endpoint was tumor width/length > 15 mm or area > 225 mm^2^ (three control mice with FaDu tumors reached experimental endpoints on day 11 and one treatment mouse with NTERA-2 tumor met humane endpoint criteria on day 20). Upon completion of the experiments, mice were euthanized with 30–40% CO_2_ vol/min flow rate, followed by cervical dislocation and visual confirmation for the absence of breathing and heartbeat to verify death, according to standard procedures at the University of Calgary.

### 2.8. Statistical Analysis

Data were presented as mean ± standard deviation (SD) from three independent experiments, unless stated otherwise. The one-way or two-way analysis of variance (ANOVA) followed by Tukey’s post hoc test was performed at a significance of *p* < 0.05. Analysis was conducted using Prism (Version 10.2, GraphPad Software, Boston, MA, USA).

## 3. Results

### 3.1. PV-10 Inhibits Growth of Diverse Adult Solid Tumor Cell Lines

**Figure 1:**
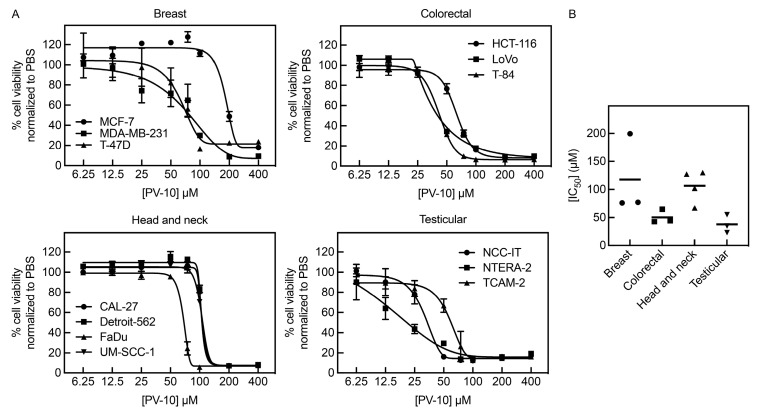
PV-10 decreases cell viability in adult solid tumor cells. (**A**) Cell lines derived from adult solid tumors were treated with increasing concentrations (6.25–400 µM) of PV-10 for 96 h. The percent cell viability was determined by alamar blue assay and normalized to PBS (vehicle control) treatment. (**B**) The mean and distribution of half maximal inhibitory concentration (IC_50_) values (μM) for breast, colorectal, head and neck, and testicular cancer cells.

To determine the effects of PV-10 on adult solid tumor cell lines with distinct molecular and phenotypic features, we treated a panel of diverse cell lines (summarized in Table S1) with concentrations of PV-10 ranging from 6.25 μM to 400 μM for 96 h, followed by cell viability measurements. PV-10 decreased cell viability in a dose-dependent manner in all cell lines tested (Figure 1A). Based on the calculated IC_50_ values for the cell lines (Table 1), testicular cancer cells were highly sensitive to PV-10 treatment (mean IC_50_ ± SD: 37.5 μM ± 16.4 μM; IC_50_ range: 23 μM–55 μM), followed by colorectal (50.4 μM ± 12.5 μM; 42 μM–65 μM), head and neck (106.6 μM ± 29.2 μM; 67 μM–130 μM), and breast cancer (117.5 μM ± 71.0 μM; 76 μM–200 μM) (Figure 1B).

### 3.2. PV-10 Induces Cancer Cell Apoptosis and Autophagy

**Figure 2:**
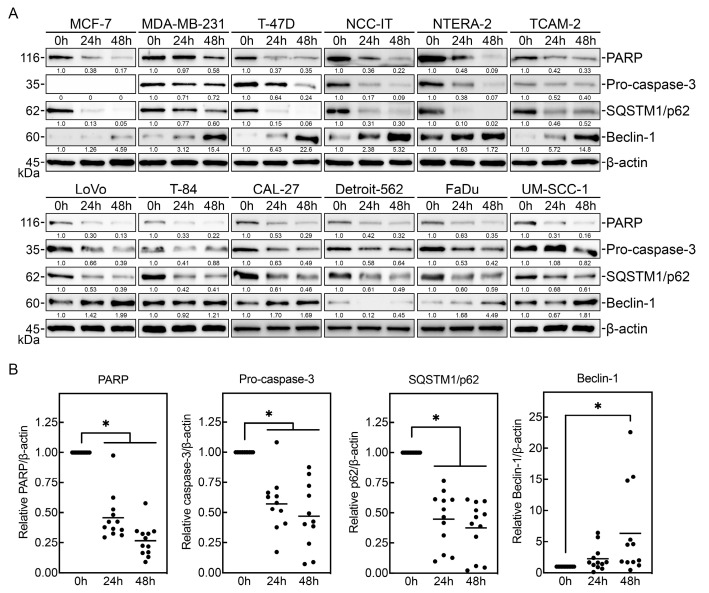
PV-10 induces apoptosis and autophagy in adult solid tumor cells. (**A**) Adult solid tumor cell lines treated with PBS (vehicle control) or 100 μM PV-10 for 24 or 48 h were immunoblotted with antibodies against PARP, pro-caspase-3, SQSTM1/p62, and beclin-1 to detect markers associated with apoptosis (decreased total PARP, increased cleaved PARP, and decreased pro-caspase-3) and autophagy (decreased SQSTM1/p62 and increased beclin-1). β-actin was used to assess protein loading. (**B**) The relative level of total PARP, pro-caspase-3, SQSTM1/p62, and beclin-1 normalized to β-actin was determined by densitometry and the mean and distribution of each cell is shown. * *p* < 0.05.

To determine whether PV-10 promotes apoptosis and autophagy in these adult solid tumor cell lines, cells were treated with 100 μM PV-10 for 24 and 48 h. Cell lysates were analyzed by Western blot to detect levels of markers associated with apoptosis and autophagy induction (Figure 2A). PV-10 treatment induced markers of apoptosis with decreased total PARP and pro-caspase-3 levels [18] in breast, colorectal, head and neck, and testicular cancer cells (Figure 2B). Furthermore, PV-10 treatment consistently led to downregulation of SQSTM1/p62 and upregulation of beclin-1 (Figure 2B), which are markers for autophagy [19,20,21]. Altogether, these data indicate that PV-10 treatment promotes time-dependent activation of drug-induced apoptosis and autophagy.

### 3.3. PV-10 Downregulates Key Phospho-Kinase Signaling Proteins

**Figure 3:**
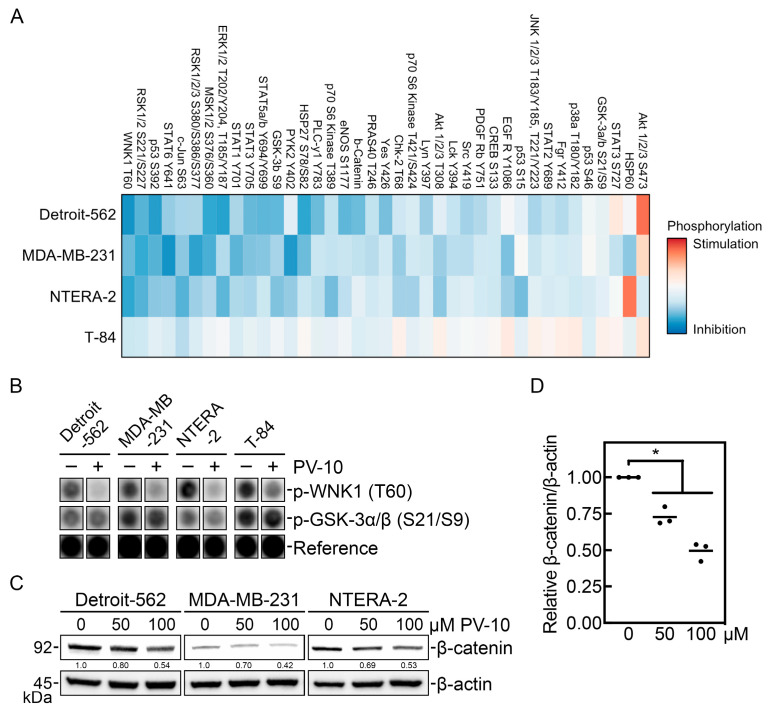
PV-10 downregulates WNK1 phosphorylation and Wnt/β-catenin signaling in adult solid tumor cells. (**A**) Detroit-562 (head and neck), MDA-MB-231 (breast), NTERA-2 (testicular), and T-84 (colorectal) cell lines treated with PBS (vehicle control) or 100 μM PV-10 for 3 h were subjected to a phospho-kinase array to determine the relative level of phosphorylation at the indicated residues for 37 proteins. The heat map plots the relative signal intensity upon PV-10 treatment compared to the control, with blue and red colors representing inhibition and stimulation, respectively. (**B**) Representative dot blots for the level of phosphorylated WNK1 (T60) and phosphorylated GSK-3α/β (S21/S9) are shown for each cell. The reference spot containing unrelated biotinylated proteins was used to assess equal development by chemiluminescence. (**C**) Detroit-562, MDA-MB-231, and NTERA-2 cells treated with PBS (vehicle control) or 50 or 100 µM PV-20 for 3 h were immunoblotted with antibodies against β-catenin and β-actin. (**D**) The relative level of β-catenin normalized to β-actin was determined by densitometry and the mean and distribution of each cell is shown. * *p* < 0.05.

Next, we investigated target modulation by PV-10 on protein kinase signaling and specific oncogenic pathways in representative cell lines from each tumor site (head and neck: Detroit-562; breast: MDA-MB-231; testicular: NTERA-2; and colorectal: T-84). Cells were treated with 100 μM PV-10 for 3 h, followed by analysis of kinase activity using an array-based assay. PV-10 treatment reduced phosphorylation of several phospho-kinase signaling proteins, including an inhibitor of autophagy, with no lysine/K (WNK) lysine deficient protein kinase 1 (WNK1) [22,23], which was also the most dysregulated candidate (Figure 3A). To validate the loss of WNK1 activity due to decreased phosphorylation of its threonine-60 (T60) residue (Figure 3B), we assessed the level of β-catenin, which is regulated by WNK1 [24,25], in cells treated with 50 µM or 100 µM PV-10 for 3 h (Figure 3C). Consistent with decreased WNK1 activity, PV-10 treatment was associated with concentration-dependent decreased levels of β-catenin (Figure 3D).

### 3.4. PV-10 Inhibits Cancer Cell Migration

**Figure 4:**
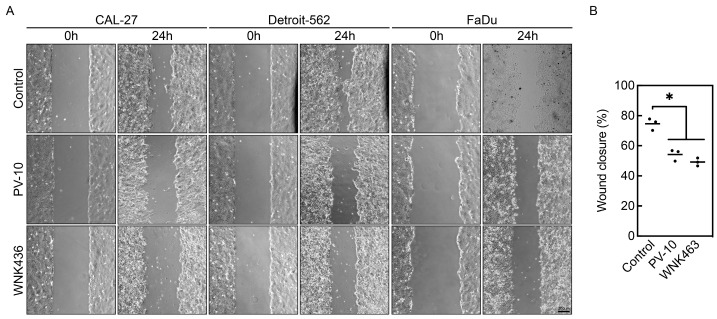
PV-10 inhibits migration of head and neck cancer cells. (**A**) Head and neck cancer cells (CAL-27, Detroit-562, and FaDu) treated with PBS (vehicle control) or sublethal doses of PV-10 (10 μM) or WNK463 (1 μM) were subjected to wound healing assays. Representative images from the start (0 h) and end point (24 h) are shown. Scale bar = 200 µm. (**B**) The percent wound closure was quantified by comparison of the area containing cells at 24 h compared to 0 h and the mean and distribution for each cell type is shown. * *p* < 0.05.

Because WNK1 regulates cell migration [26,27,28] and β-catenin signaling also promotes metastasis in head and neck cancer [29,30], wound healing assays were performed with sublethal doses of PV-10 to investigate its effect on migration of aggressive head and neck cancer cells (Figure 4A). Treatment of 10 µM PV-10 significantly inhibited migration of CAL-27, Detroit-562, and FaDu cells, compared to the control (Figure 4B). A similar extent of cell migration inhibition was observed in cells exposed to a pan-WNK kinase inhibitor, WNK463 [31]. These results indicate that interfering with WNK1 and β-catenin, either by treatment with PV-10 or WNK463, reduces migration of invasive types of adult solid tumor cells.

### 3.5. Intralesional and Systemic Administration of PV-10 Decreases Tumor Growth In Vivo

**Figure 5:**
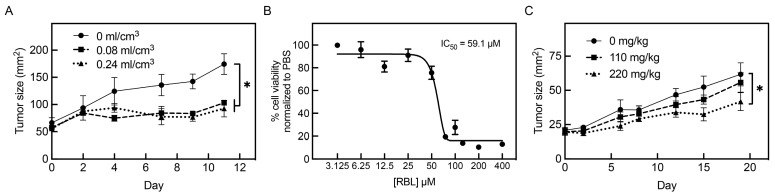
PV-10 decreases tumor growth in vivo by intralesional or oral administration. (**A**,**C**) CD17 SCID mice carrying FaDu (**A**) or NTERA-2 (**C**) tumors were treated with intralesional PV-10 or oral RBL, respectively. For intralesional injections (**A**), mice were treated with 0.08 or 0.24 mL of PV-10 per cm^3^ lesion volume (mL/cm^3^). For oral gavage (**C**), mice were treated twice weekly with 110 or 220 mg of RBL per kg of body weight (mg/kg). Control mice received an equivalent volume of PBS via the same route of administration. Tumor size (mm^2^) was regularly monitored by vernier caliper measurements. Values represent means ± SD from 3–6 mice per treatment group. * *p* < 0.05. (**B**) NTERA-2 cells were treated with increasing concentrations (3.125–400 µM) of RBL for 96 h. The percent cell viability was determined by alamar blue assay and normalized to PBS (vehicle control) treatment.

To investigate if PV-10 is also effective in vivo, we characterized the effect of treatment in CB17 SCID mice with subcutaneous FaDu and NTERA-2 xenograft tumors. Mice carrying FaDu tumors responded to intralesional PV-10 with decreased tumor growth (Figure 5A). For control mice with FaDu tumors, mean tumor size increased 108 mm^2^ from 66.5 mm^2^ to 174.5 mm^2^ in 11 days, reaching the experimental endpoint. By comparison, the size of tumors treated with 0.08 and 0.24 mL/cm^3^ of intralesional PV-10 only increased by 45 and 38 mm^2^, respectively. Next, we tested systemic administration with RBL, a lipophilic derivative of PV-10 that induced similar cytotoxicity against NTERA-2 cells in vitro (Figure 5B) and demonstrated systemic tolerability in vivo, without specific pathology identified upon oral treatment in normal mice (Table S2). After implantation, mice bearing NTERA-2 tumors were treated twice weekly with 110 or 220 mg/kg of RBL via oral gavage. RBL significantly reduced tumor size at 220 mg/kg (Figure 5C). For control NTERA-2 tumors, the tumor size increased 41 mm^2^ from 20.8 mm^2^ to 61.8 mm^2^ 19 days post-treatment. By comparison, the size of tumors treated with 110 and 220 mg/kg of oral RBL only increased by 36 and 23 mm^2^, respectively. In summary, PV-10 and RBL inhibit in vivo growth of adult solid tumor cells in a dose-dependent manner by intralesional and systemic administration, respectively.

## 4. Discussion

In both preclinical and clinical studies, the active ingredient in PV-10, RBS, has previously demonstrated potent anti-tumor activity by intralesional injection for several types of cancer, including skin, breast, and colon tumors. However, systemic administration of PV-10 for the treatment of solid tumors has not been tested. To explore the use of PV-10 for the treatment of solid tumors, we initially tested the drug in a panel of solid tumor cell lines derived from male and female adults with breast, colorectal, head and neck, and testicular cancers. We found that PV-10 decreased cell viability of these cancer cells in a concentration-dependent manner. The IC_50_ values achieved by PV-10 in our study were similar to other reports studying RBS, with cytotoxicity observed in the same range for colon, gastric, neuroblastoma, and ovarian cancer cell lines [14,15,16,17]. Furthermore, there is an appreciable therapeutic window in comparison to the cytotoxicity of PV-10 observed in normal fibroblast cells [15,16,32]. Our results indicate that PV-10 induces cytotoxicity in a broad range of adult solid tumor cells.

To further characterize PV-10-mediated cytotoxicity, we next investigated the mechanism of cell death. Previous studies have shown that RBS and PV-10 primarily induce cell death by apoptosis [14,15,16,17,32]. Our analysis revealed a decrease in total PARP, likely due to caspase-mediated cleavage, upon treatment with PV-10, indicating drug-induced apoptosis [18]. Accordingly, the level of pro-caspase-3 was also reduced in all PV-10-treated cells except MCF-7, which do not express caspase-3 [33,34], likely due to the activation of cleaved caspase-3. Based on PV-10-induced lysosome degradation [15] and its role in autophagic cell death [35], we also posited the potential for autophagy-induced cell death. Remarkably, PV-10-treated cells had consistent degradation of SQSTM1/p62, which is related to autophagic flux [19]. Additionally, PV-10-treated cells also upregulated expression of beclin-1, which contributes to autophagic cell death [21,36]. In some cells, conversion of LC3B-I to LC3B-II was also noted; however, LC3 levels may not always accurately represent autophagic flux due to degradation of LC3B-II itself during autophagy [20]. Altogether, our data suggest that PV-10 induces cell death by a combination of apoptotic and autophagic pathways.

Next, we investigated the effect of target modulation of PV-10 on signaling in specific oncogenic pathways. Using an array-based assay, we observed that short-term exposure to PV-10 downregulates protein kinase activity. Notably, the most dysregulated kinase in the screen was WNK1, which may explain the PV-10-induced autophagy due to inhibition of autophagy by WNK1 [22,23]. WNK1 also regulates Wnt signaling through β-catenin [24,25], which was decreased in PV-10-treated cells with less activity of WNK1. WNK1 has been implicated in tumor cell migration and invasive characteristics in breast [26,27], lung [37], and prostate [38] cancers. Inhibition of WNK signaling using the allosteric inhibitor WNK463 [31] has been shown to decrease epithelial–mesenchymal markers and reduce invasive potential [27], which is also observed with the knockdown of WNK1 [26,28,37]. We show that treating cells with PV-10 or WNK463 significantly reduces the migratory capacity of tumor cells in a similar fashion. WNK1 also regulates Wnt signaling by preventing degradation of β-catenin and inhibition of WNK signaling prevented tumor growth in mice [25]. Consequently, inhibition or loss of β-catenin attenuates cell migration and invasive properties of head and neck cancer cells [29,30]. Our data provide evidence of PV-10-mediated downregulation of signaling pathways, including WNK1 and Wnt/β-catenin signaling.

Having determined that PV-10 is cytotoxic to adult solid tumor cell lines in vitro, we then tested in vivo activity using subcutaneous head and neck and testicular tumor xenografts in mice. Because these experiments primarily aimed to evaluate the effect of PV-10, all tumors were generated in female mice for practical reasons to minimize confounders. Consistent with clinical studies testing PV-10 by intralesional injection [6,7,8,9,10,11], we found that pharmacologically relevant doses of intralesional PV-10 induced significant tumor growth inhibition in immunodeficient mice with FaDu tumors. Further supporting tumor regression, PV-10-treated mice had reduced average tumor masses than control mice. Uniquely, we also discovered that RBL, a lipophilic derivative of PV-10 to enhance oral bioavailability, has anti-tumor activity when delivered systemically. NTERA-2-bearing mice orally treated with RBL had significantly reduced tumor growth and a trend towards improved survival. Because PV-10 has been shown to induce anti-tumor immune responses and immunogenic cell death [13,14], we anticipate that the effect of PV-10 would be augmented in an immunocompetent model. Demonstrating systemic tolerability, analysis of hematoxylin and eosin staining by an independent pathologist found no evidence of specific pathology or significant acute or chronic inflammation in the brain, heart, kidneys, liver, lungs, or spleen of normal mice orally treated with increasing concentrations of RBL for two weeks. Several studies have established that intralesional treatment with PV-10 also improves the response at distant non-target lesions [6,9,13], highlighting the importance of understanding systemic responses to PV-10. Future studies should further evaluate the effect of various doses through systemic administration in combination with standard therapies.

## 5. Conclusions

In summary, this study provides preclinical proof-of-concept data supporting the efficacy of PV-10 in a broad panel of adult solid tumors. Mechanistically, we identify that PV-10 downregulates WNK1 and Wnt signaling through the loss of β-catenin, ultimately inhibiting cell migration and inducing cell death through both apoptotic and autophagic pathways. Finally, we demonstrate the clinical utility of PV-10 and RBL in vivo by intralesional or systemic administration, a novel route of administration, in tumor xenograft mouse models. In conclusion, our data provide essential mechanism-based evidence and biomarkers of activity to support further clinical studies of PV-10 in cancer treatment protocols.

## Figures and Tables

**Table 1:** Half-maximal inhibitory concentrations (IC50) for adult solid tumor cell lines treated with PV-10.

Tumor	Cell Line	IC_50_ (μM)
Breast	MCF-7	199.5
	MDA-MB-231	76.99
	T-47D	75.98
Colorectal	HCT-116	64.79
	LoVo	43.96
	T-84	42.37
Head and neck	CAL-27	127.3
	Detroit-562	129.9
	FaDu	67.09
	UM-SCC-1	102.0
Testicular	NCC-IT	34.54
	NTERA-2	22.77
	TCAM-2	55.13

## Data Availability

The data presented in this study are available upon request from the corresponding author.

## Supplementary Materials

The following supporting information can be downloaded at: https://www.mdpi.com/article/10.3390/cancers16081520/s1. Table S1. Characteristics of the adult solid tumor cell lines used in this study. Table S2. Pathology report of tissue isolated from normal mice orally treated with rose bengal. File S1. Original Western blot figures.
