# Supplementary material for: Identification and In Vivo Validation of Unique Anti-Oncogenic Mechanisms Involving Protein Kinase Signaling and Autophagy Mediated by the Investigational Agent PV-10
Source: Cancers (Basel). 2024 Apr 16;16(8):1520. doi: 10.3390/cancers16081520 (PMC11048188; doi:10.3390/cancers16081520)
Supplement: Supplementary file 1 [file cancers-16-01520-s001.zip › cancers-2926326-supplementary.pdf]

Table S1. Characteristics of the adult solid tumor cell lines used in this study.

Tumor	Cell line	Age/sex	Genetic abnormalities
Breast	MCF-7	69/F	CDKN2A Hom Del; GATA3 Het N336Mfs*17; PIK3CA Het E545K; Lacks TP53 mutation
	MDA-MB-231	51/F	Triple negative; BRAF Het G464V; CDKN2A Del; KRAS Het G13D; TERT C228T; TP53 Hom R280K
	T-47D	54/F	PIK3CA Het H1047R; TP53 Hom L194F
Colorectal	HCT-116	48/M	ACVR2A Hom K437Rfs*5; BRCA2 Het I2675Dfs*6; CDKN2A Het R24fs*20 and E74fs*15; CTNNB1 Het S45del; EP300 Hem c.6294delA; KRAS Het G13D; PIK3CA Het H1047R; TGFBR2 Hom K128Sfs*35; Lacks TP53 mutation
	LoVo	56/M	ACVR2A Hom K437Rfs*5; APC Het R114Ter; APC Het M1431fs*42; APC Het R2816Q; B2M Het L15Ffs*41; FBXW7 Het R505C; KRAS Het G13D; SMAD2 Het A292V; TGFBR2 Hom K128Sfs*35; Lacks TP53 mutation
	T-84	72/M	APC Hom L1488fs*19; KRAS Het G13D; PIK3CA E542K; SMAD4 K340N
Head and neck	CAL-27	56/M	TERT c.228C>T; TP53 H193L
	Detroit-562	*/F	TP53 R175H
	FaDu	56/M	CDKN2A Hom c.151-1G>T; TP53 Het R248L and Y126_K132del
	UM-SCC-1	72/M	Lacks TP53 mutation
Testicular	NCC-IT	24/M	MAP2K4 K309N; TP53 Hom V272Cfs*73; PTEN Het R173P
	NTERA-2	22/M	High TP53 expression but lowered p53 activity due to K370me1 and K382me1 monomethylation
	TCAM-2	35/M	BRAF V600E; Lacks CD30, SSX2-4, and SOX2 expression

* Age/sex unknown

Table S2. Pathology report of tissue isolated from normal mice orally treated with rose bengal.

Tissue	Specimen A (0 mg/kg)	Specimen B (50 mg/kg)	Specimen C (100 mg/kg)	Specimen D (200 mg/kg)
Brain	No specific pathology	No specific pathology	No specific pathology	No specific pathology
Heart	No specific pathology	No specific pathology	No specific pathology	No specific pathology
Kidney	Malignant neoplasm identified: Perinephric tumor, cytologically compatible with lymphoid origin	No specific pathology identified in renal parenchyma; Adrenal tissues also identified, without specific pathology	No specific pathology identified in renal parenchyma	No specific pathology identified in renal parenchyma; Adrenal tissues also identified, without specific pathology
Liver	No specific pathology	No specific pathology	No specific pathology	No specific pathology
Lung	No specific pathology	No specific pathology	No specific pathology	No specific pathology
Spleen	No specific pathology; Pancreatic tissues also identified, without specific pathology	No specific pathology; Pancreatic tissues also identified, without specific pathology	No specific pathology; Pancreatic tissues also identified, without specific pathology	No specific pathology; Pancreatic tissues also identified, without specific pathology

Figure S2A

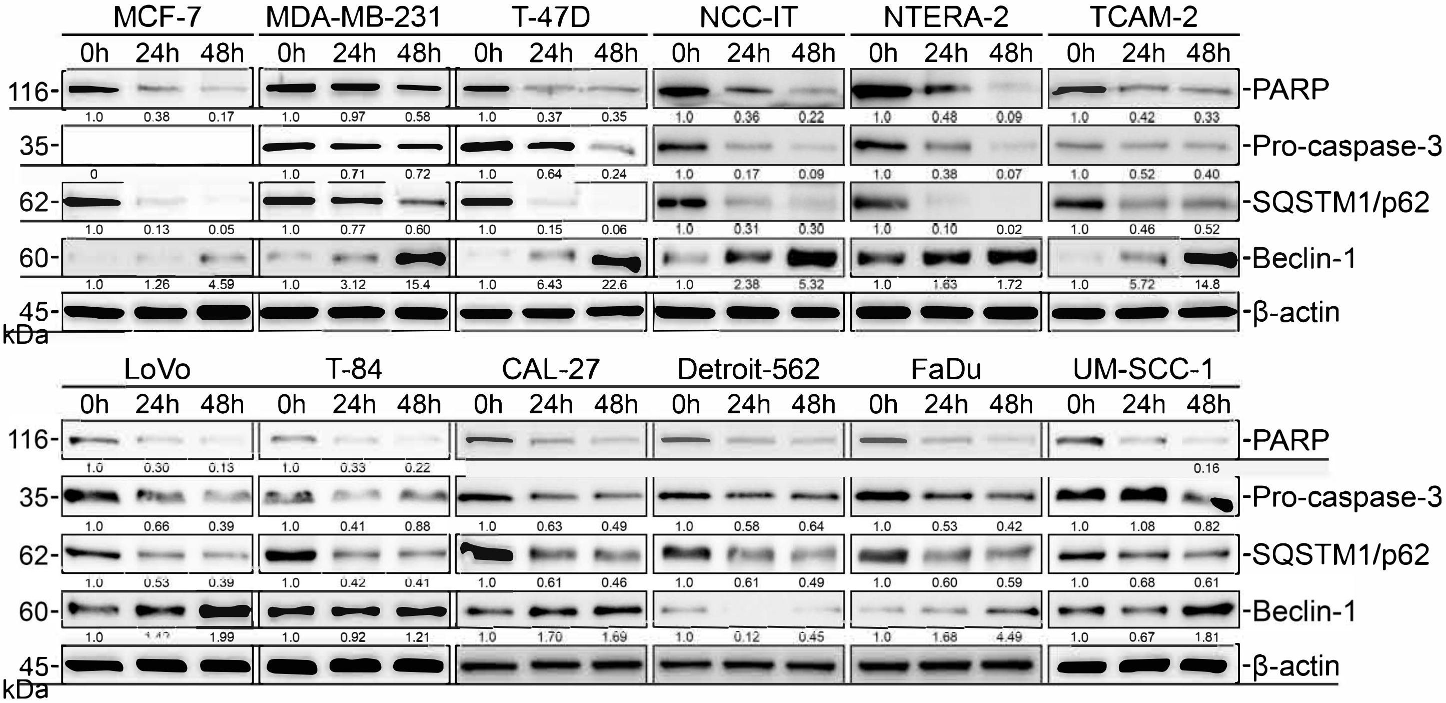

Figure S3C

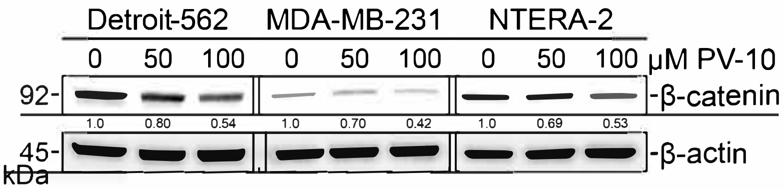

Figure S2A

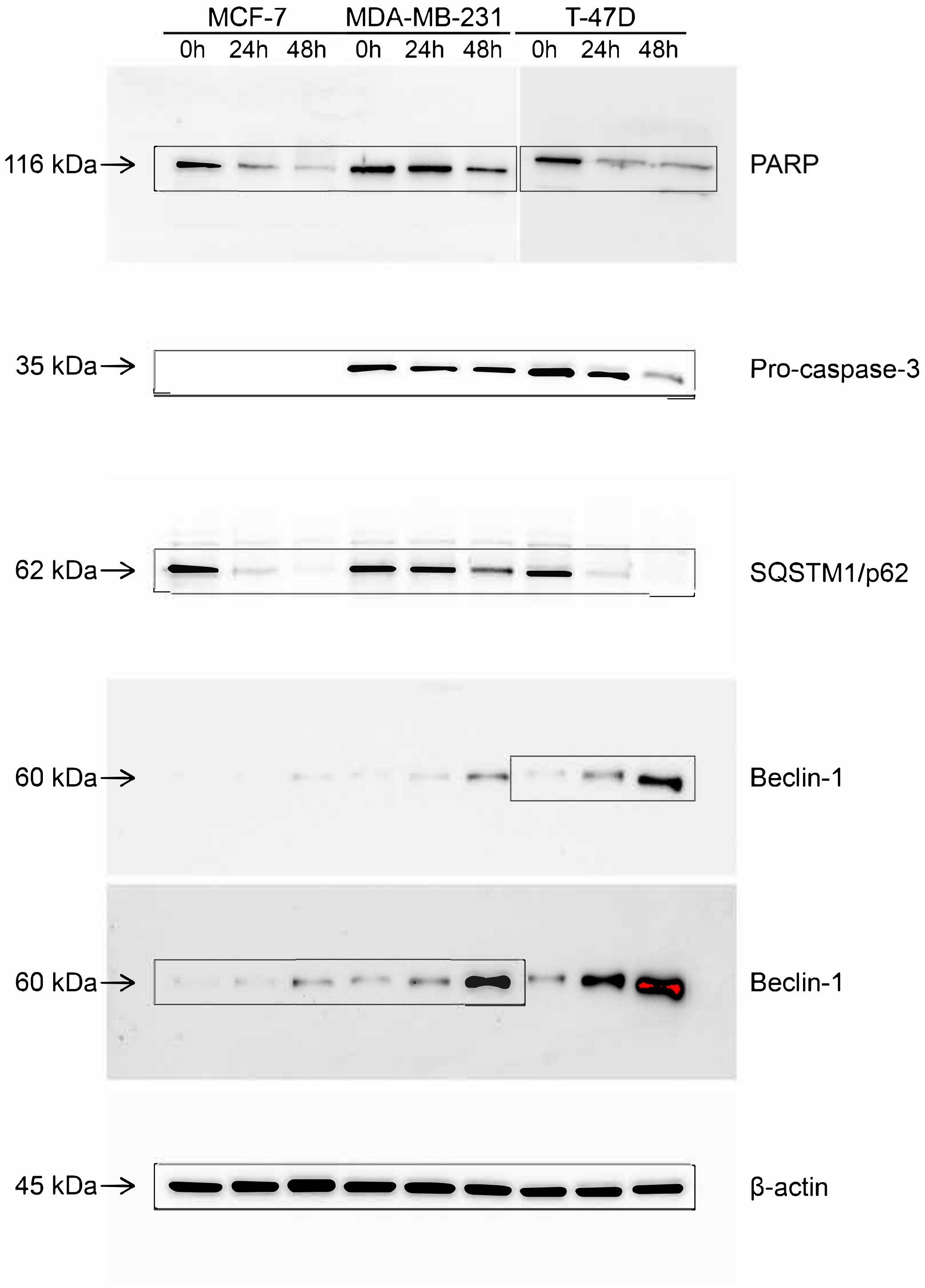
MCF-7
MDA-MB-231
T-47D
0h 24h 48h 0h 24h 48h 0h 24h 48h
116 kDa
PARP
35 kDa
Pro-caspase-3
62 kDa
SQSTM1/p62
60 kDa
Beclin-1
60 kDa
Beclin-1
45 kDa
β-actin

Figure S2A

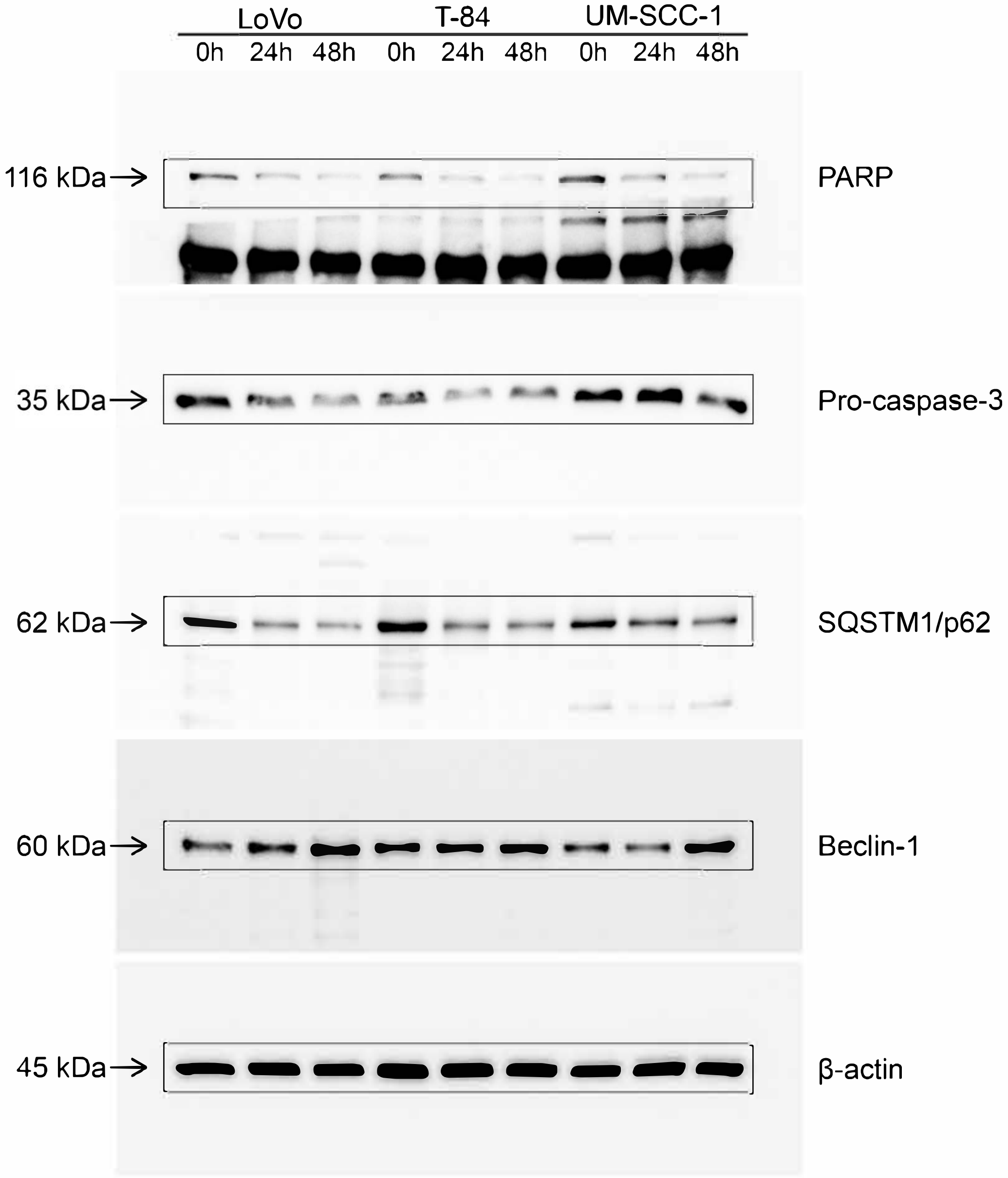

LoVo
T-84
UM-SCC-1
0h 24h 48h 0h 24h 48h 0h 24h 48h
116 kDa
PARP
35 kDa
Pro-caspase-3
62 kDa
SQSTM1/p62
60 kDa
Beclin-1
45 kDa
β-actin

Figure **S**2A

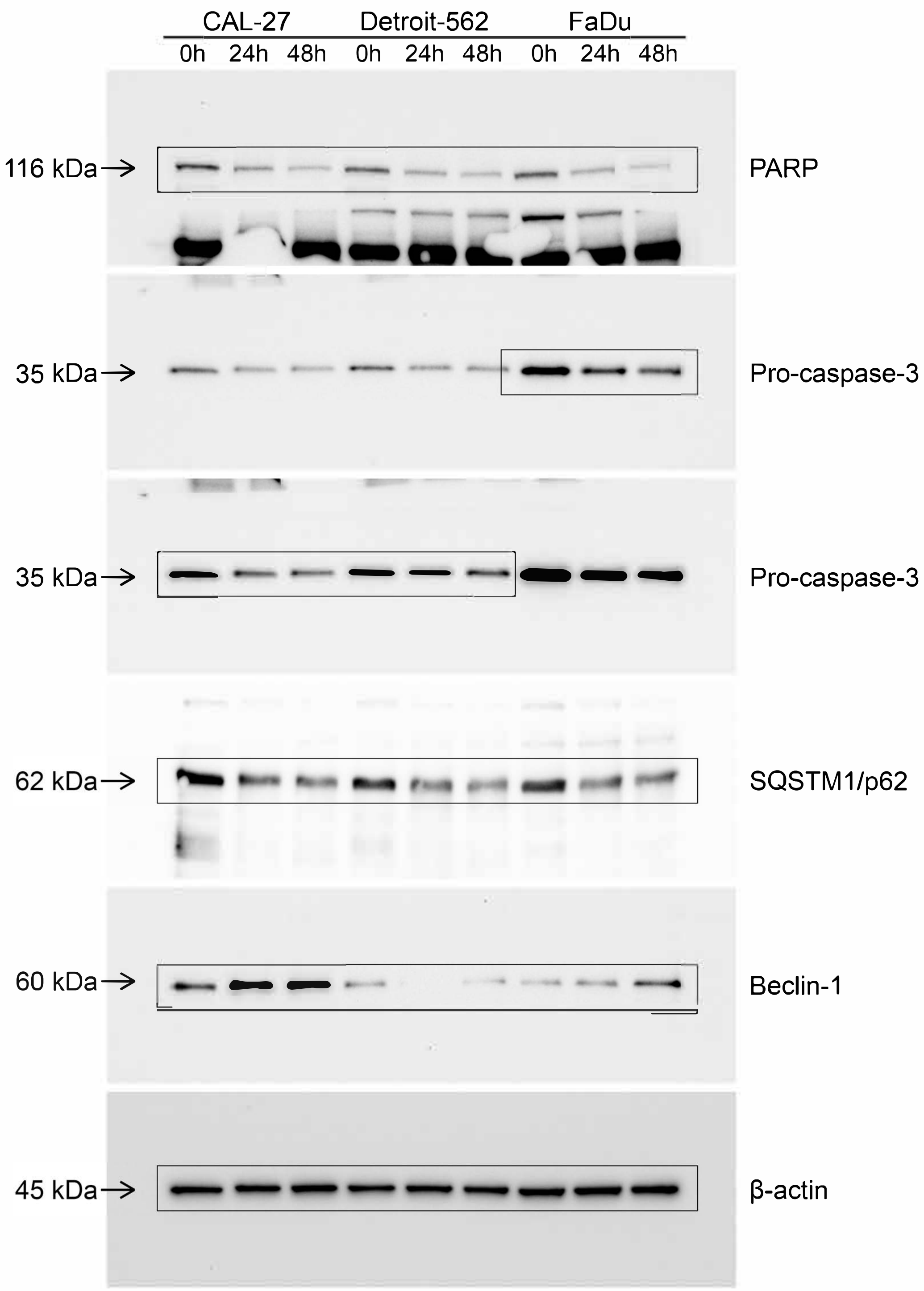
CAL-27
Detroit-562
FaDu
0h 24h 48h 0h 24h 48h 0h 24h 48h
116 kDa
PARP
35 kDa
Pro-caspase-3
35 kDa
Pro-caspase-3
62 kDa
SQSTM1/p62
60 kDa
Beclin-1
45 kDa
β-actin

Figure S2A

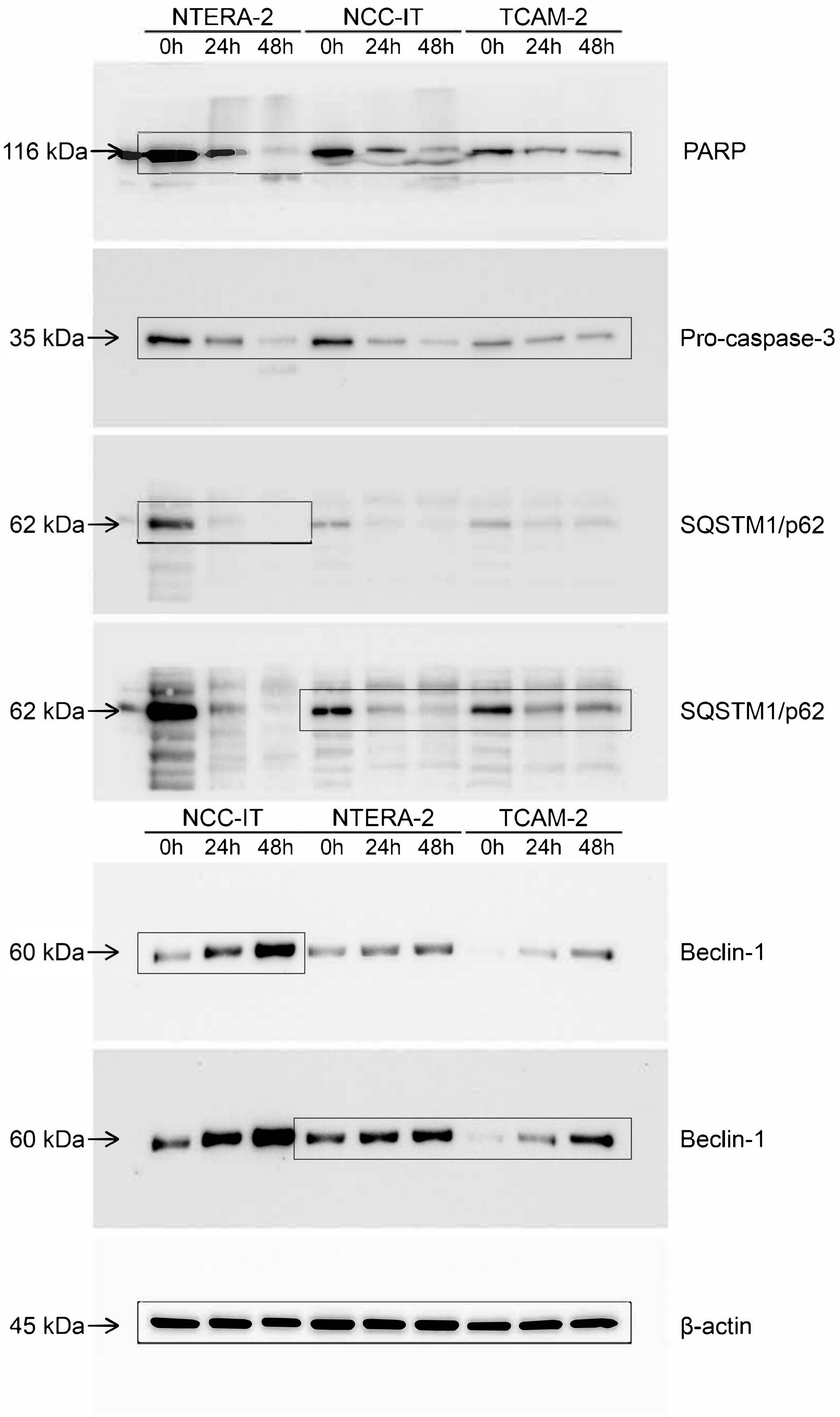

NTERA-2
NCC-IT
TCAM-2
0h
24h
48h
0h
24h
48h
0h
24h
48h
116 kDa
PARP
35 kDa
Pro-caspase-3
62 kDa
SQSTM1/p62
62 kDa
SQSTM1/p62
NCC-IT
NTERA-2
TCAM-2
0h
24h
48h
0h
24h
48h
0h
24h
48h
60 kDa
Beclin-1
60 kDa
Beclin-1
45 kDa
β-actin

Figure S3B

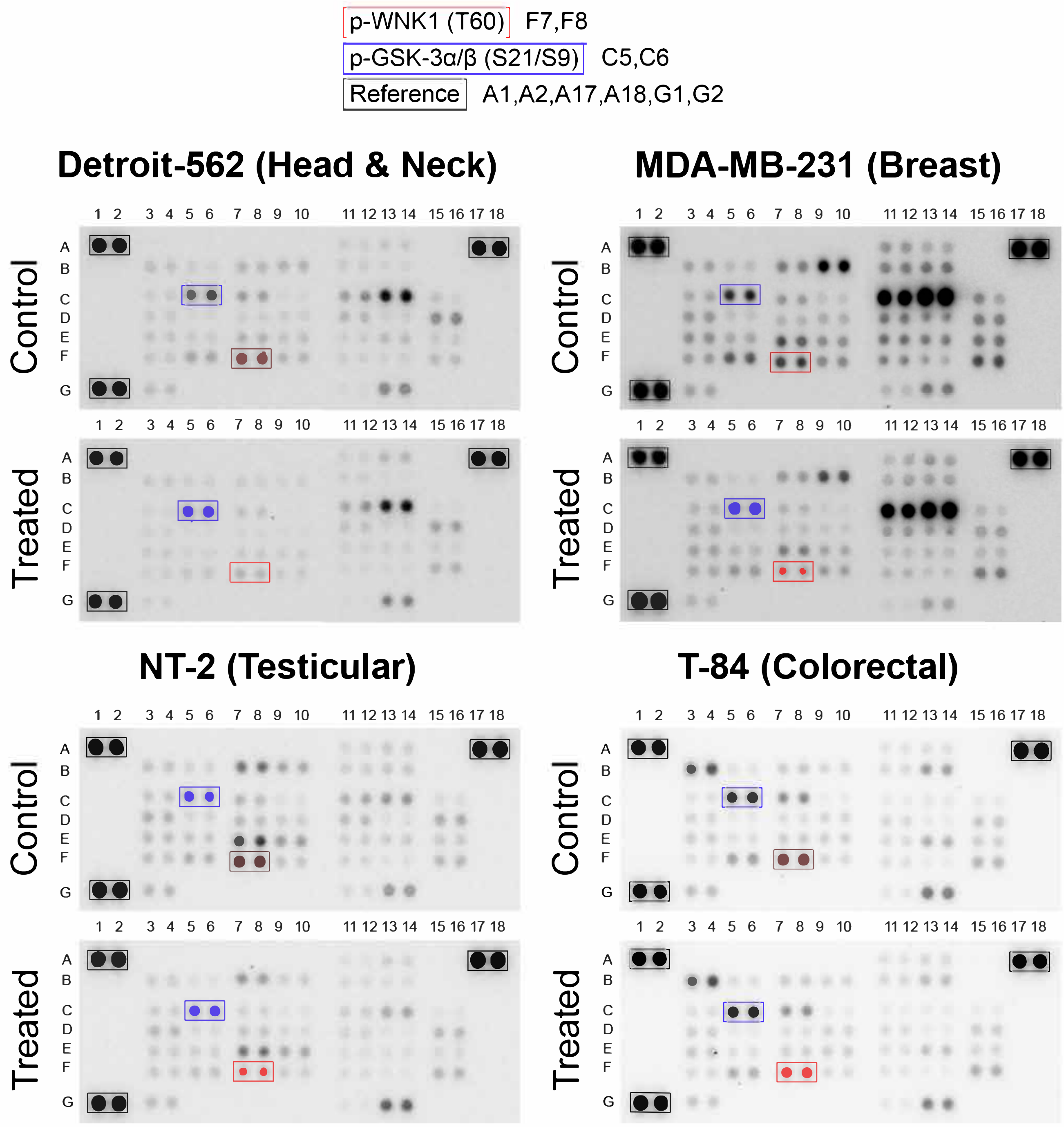
p-WNK1 (T60) F7,F8
p-GSK-3α/β (S21/S9) C5,C6
Reference A1,A2,A17,A18,G1,G2
Detroit-562 (Head & Neck)
MDA-MB-231 (Breast)
NT-2 (Testicular)
T-84 (Colorectal)
Control
Treated
1 2 3 4 5 6 7 8 9 10 11 12 13 14 15 16 17 18
A
B
C
D
E
F
G

Figure S3C

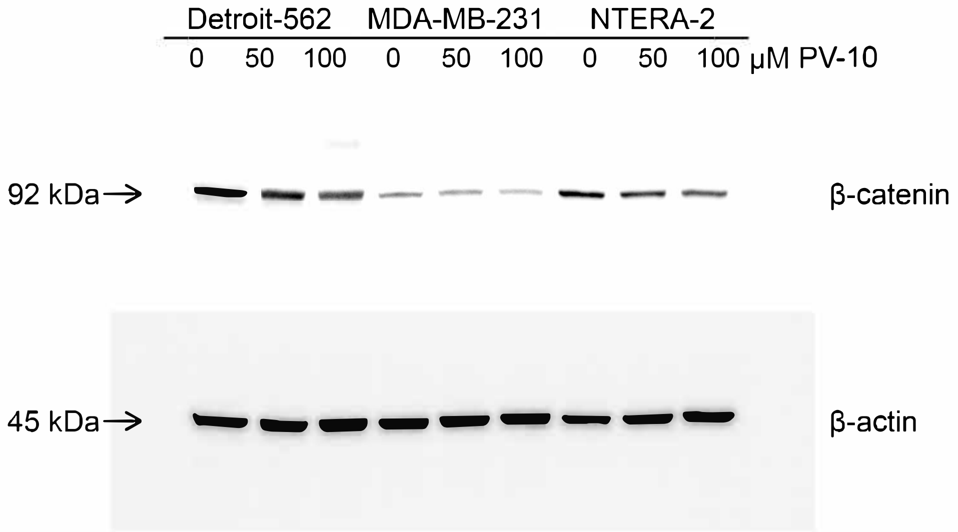

Detroit-562 MDA-MB-231 NTERA-2
0 50 100 0 50 100 0 50 100 µM PV-10
92 kDa
β-catenin
45 kDa
β-actin

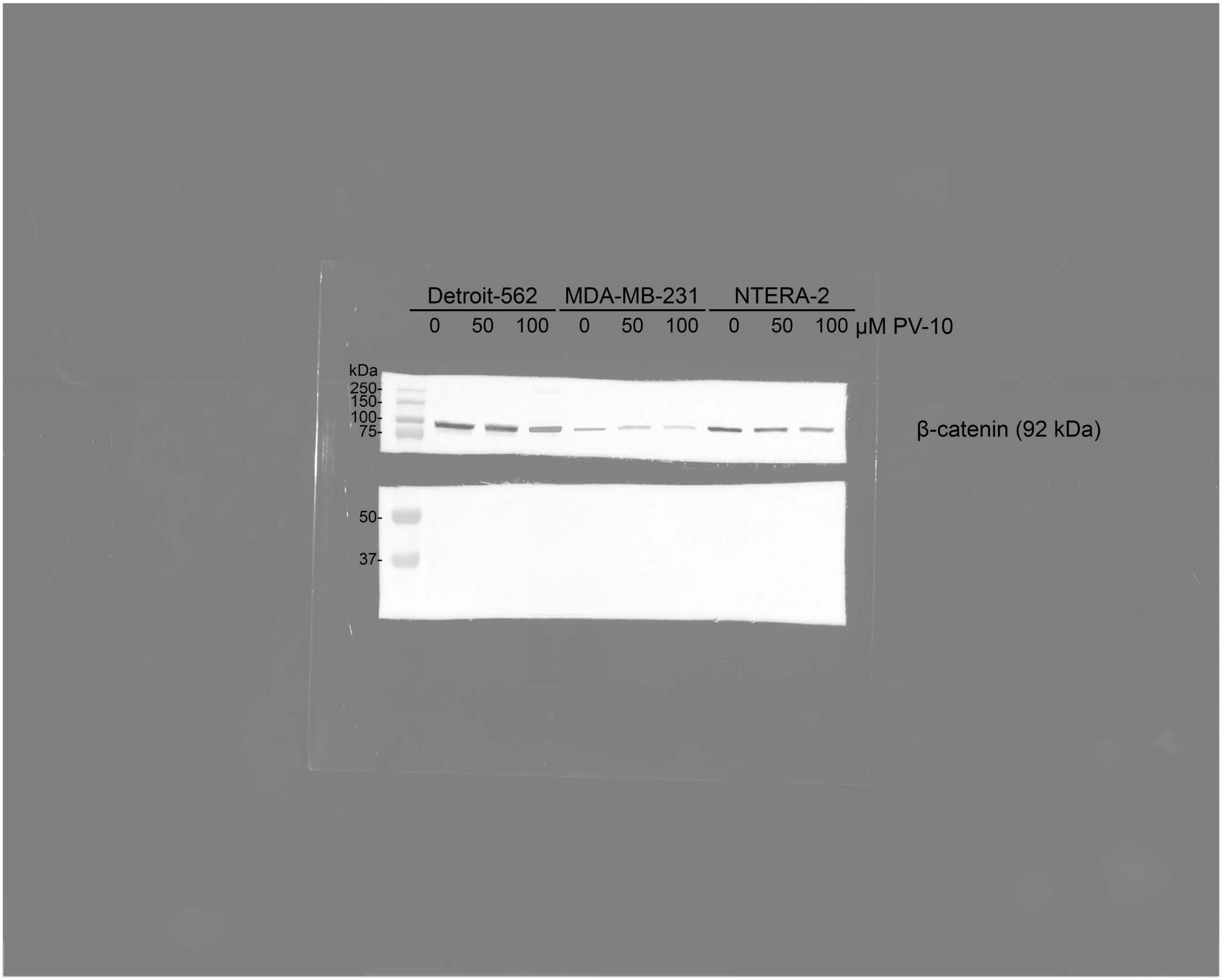

Detroit-562 MDA-MB-231 NTERA-2
0 50 100 0 50 100 0 50 100 µM PV-10
kDa
250-
150-
100-
75-
β-catenin (92 kDa)
50-
37-
